# Fos expression in the prefrontal cortex and ventral striatum after exposure to a free-operant timing schedule

**DOI:** 10.1016/j.bbr.2012.08.009

**Published:** 2012-12-01

**Authors:** L. Valencia-Torres, C.M. Olarte-Sánchez, S. Body, T.H.C. Cheung, K.C.F. Fone, C.M. Bradshaw, E. Szabadi

**Affiliations:** a Psychopharmacology Section, Division of Psychiatry, University of Nottingham, UK; b School of Biomedical Sciences, University of Nottingham, UK

**Keywords:** Orbital prefrontal cortex, Nucleus accumbens, Ventral striatum, Fos expression, Interval timing behaviour, Temporal differentiation, Free-operant psychophysical procedure, Variable-interval schedule, Rat

## Abstract

It has been proposed that cortico-striato-thalamo-cortical circuits that incorporate the prefrontal cortex and corpus striatum regulate interval timing behaviour. In the present experiment regional Fos expression was compared between rats trained under an immediate timing schedule, the free-operant psychophysical procedure (FOPP), which entails temporally regulated switching between two operanda, and a yoked variable-interval (VI) schedule matched to the timing task for food deprivation level, reinforcement rate and overall response rate. The density of Fos-positive neurones (counts mm^−2^) in the orbital prefrontal cortex (OPFC) and the shell of the nucleus accumbens (AcbS) was greater in rats exposed to the FOPP than in rats exposed to the VI schedule, suggesting a greater activation of these areas during the performance of the former task. The enhancement of Fos expression in the OPFC is consistent with previous findings with both immediate and retrospective timing schedules. Enhanced Fos expression in the AcbS was previously found in retrospective timing schedules based on conditional discrimination tasks, but not in a single-operandum immediate timing schedule, the fixed-interval peak procedure. It is suggested that the ventral striatum may be engaged during performance on timing schedules that entail operant choice, irrespective of whether they belong to the immediate or retrospective categories.

## 1 Introduction

“Interval timing” refers to the ability of organisms to adapt their behaviour to temporal regularities in their environments. Many types of reinforcement schedule have been devised to assess interval timing in animals. Killeen and Fetterman [1,2] developed a taxonomy of these schedules based on the relationship between the animal's timing response and the interval being timed. According to this taxonomy, the three main classes of timing schedule are (i) *retrospective* timing schedules, in which the subject is trained to emit different responses depending upon the duration of an interval that has already elapsed when the response is made (temporal discrimination), (ii) *immediate* timing schedules, in which the subject's behaviour comes under the control of time during an ongoing interval (temporal differentiation), and (iii) *prospective* timing schedules, in which the animal is trained to emit discriminative responses on the basis of intervals that follow the responses (inter-temporal choice).

A growing body of evidence supports the involvement of cortico-striato-thalamo-cortical circuits in interval timing [3,4]. For instance, the firing rate of striatal and cortical neurones has been found to track timing performance in immediate timing schedules [5,6], and lesions of the dorsal striatum in rats have been found to disrupt timing on these schedules [7]. Electrophysiological and functional imaging studies in humans have also demonstrated that the dorsal striatum is activated during performance of some temporal discrimination tasks [8–10].

Recent findings by Valencia-Torres et al. [11] suggest that the ventral striatum (nucleus accumbens) may play a significant role in the performance of rats on some timing tasks. These authors examined Fos expression, a marker of neuronal activation, in the prefrontal cortex and corpus striatum following performance on retrospective timing schedules (the interval bisection task [12,13] and another discrete-trials psychophysical procedure [14]). It was found that rats trained under these schedules showed enhanced Fos expression in the prefrontal cortex and ventral striatum, compared to rats trained under non-temporal (light-intensity) discriminaton tasks, indicating that these structures were activated during performance on the timing schedules. More recently, however, the same authors [15] found no significant enhancement of Fos expression in the ventral striatum following performance on an immediate timing schedule, the fixed-interval peak procedure [12,16].

The different findings of these two experiments suggest that the ventral striatum may not be equally involved in all types of interval timing performance. One possibility is that the ventral striatum may play a significant role in temporal discrimination in retrospective timing schedules but have little involvement in temporal differentiation in immediate timing schedules [15]. However, there are a number of other important differences between the fixed-interval peak procedure and the retrospective timing tasks used by Valencia-Torres et al. [11] apart their membership of different classes of timing schedule, and it is possible that these differences were more important than class membership in determining the differential involvement of the ventral striatum in different timing tasks. One such difference relates to the type of operant performance used to assess timing. In the retrospective timing tasks used by Valencia-Torres et al. [11], timing is assessed from the probability of emission of discrete responses, whereas in the fixed-interval peak procedure it is assessed from variation in the rate of free-operant responding during the course of a trial. Another difference concerns the number of operanda employed in the timing tasks. The retrospective timing tasks used by Valencia-Torres et al. [11] entailed choice between two operanda whereas the fixed-interval peak procedure entails responding on a single operandum.

The present experiment was an attempt to shed further light on the factors that may underlie the different patterns of Fos expression seen following exposure to different types of timing task [11,15]. The timing task used in this experiment was an immediate timing schedule, the free-operant psychophysical procedure (FOPP) [17,18]. In this schedule, reinforcement is provided intermittently for responding on two operanda, A and B, responding on A being reinforced in the first half, and responding on B in the second half of each trial. Temporal differentiation is assessed quantitatively from the psychometric function relating relative response rate on B (%*B*) to time measured from the onset of the trial. Thus the FOPP, like the fixed-interval peak procedure, is an immediate timing schedule that entails repetitive, free-operant responding. However, like the retrospective timing tasks used by Valencia-Torres et al. [11], and unlike the fixed-interval peak procedure, the FOPP entails choice between two simultaneously presented operanda, timing being assessed from the relative allocation of responses to the two operanda.

In the present experiment, as in the previous experiments [11,15], the pattern of Fos expression in the prefrontal cortex and corpus striatum was assessed following exposure to the timing task. Fos is the protein product of the immediate-early gene *c-fos* which is found in neuronal nuclei. In most neurones, Fos levels are low under basal conditions, but neuronal firing results in an increase in production of the protein [19]. Changes in Fos expression may therefore act as a biomarker for relatively short-term changes in neuronal activity induced by physiological or behavioural manipulations [20,21]. However, since Fos expression can be induced by various aspects of operant behavioural tasks that are not directly related to the process of primary interest, for example, food deprivation, food consumption [22,23] and locomotor activity [23–25], it is important that any experiment in which Fos expression is used as a means of identifying the neural structures involved in particular behavioural processes should employ a control procedure that is matched as closely as possible to the index task on these ‘irrelevant’ variables. In the present experiment, the pattern of Fos expression in the prefrontal cortex and corpus striatum was compared between a group of rats trained under the FOPP and a control group trained under a variable-interval (VI) schedule [26,27] presented on two levers, yoked to the FOPP. Thus the rats trained under the VI schedule received the same exposure to food deprivation, the same response requirements and the same rate of reinforcement as those trained under the FOPP. However, the distribution of responding on the two levers in the FOPP reflected temporal differentiation, whereas in the yoked VI schedule it was determined by the behaviour of ‘master’ rats trained under the FOPP. It was therefore assumed that temporal differentiation was not involved in performance on the yoked VI schedule.

## 2 Methods

The experiment was carried out in accordance with UK Home Office regulations governing experiments on living animals, and was approved by the University of Nottingham Ethical Review Committee.

### 2.1 Subjects

Twenty-four experimentally naive female Wistar rats (Charles River, UK) aged approximately 4 months and weighing 250–300 g at the start of the experiment were used. They were housed individually under a constant cycle of 12 h light and 12 h darkness (light on 06:00–18:00 h), and were maintained at 80% of their initial free-feeding body weights throughout the experiment by providing a limited amount of standard rodent diet after each experimental session. Tap water was freely available in the home cages.

### 2.2 Apparatus

The rats were trained in operant conditioning chambers of internal dimensions 20 cm × 23 cm × 22.5 cm (Campden Instruments Ltd., UK). One wall of the chamber contained a recess into which a motor-operated dipper could deliver 50 μl of a 0.6 M sucrose solution. Apertures were situated 5 cm above and 2.5 cm on either side of the recess; motor-operated retractable levers could be inserted into the chamber through these apertures. The levers could be depressed by a force of approximately 0.2 N. The chamber was enclosed in a sound-attenuating chest; masking noise (approximately 80 dB[A]) was provided by a rotary fan. An Acorn 600 microcomputer and interface unit (Paul Fray Ltd., UK), programmed in ARACHNID BASIC and located in an adjoining room, controlled the schedules and recorded the behavioural data.

### 2.3 Behavioural training

The rats were allocated to two groups that were trained under the FOPP (*n* = 12) or a yoked VI schedule (*n* = 12). Each rat in the yoked VI group was paired with a rat in the FOPP group. At the start of the experiment, the food-deprivation regimen was started and the rats were gradually reduced to 80% of their free-feeding body weights. They were then trained to press the levers by providing reinforcers intermittently, in the absence of the levers, for three sessions (50 reinforcers per session), followed by three sessions of exposure to a discrete-trials continuous reinforcement schedule, in which each lever was presented intermittently, and a single response resulted in retraction of lever and delivery of the reinforcer. Thereafter, the rats underwent 50-min training sessions on the FOPP or yoked VI schedule, as described below, 7 days a week at the same time each day during the light phase of the daily cycle (between 08:00 and 13:00 h), for >90 sessions.

*FOPP* (*n* = 12). The free-operant psychophysical procedure was the same as that used by Chiang et al. [28–30]. Each session consisted of 50 50-s trials, successive trials being separated by 10-s intertrial intervals. In 46 of the 50 trials (the ‘tandard’ trials), reinforcement was provided on a constant-probability VI 30-s schedule (constant-probability VI schedules minimize the correlation between the probability of reinforcement and time since the last reinforcement; the purpose of this specification is to prevent time since reinforcement from acquiring discriminative control over respondings. Performance on constant-probability VI schedules is characterized by local response rates that show minimal variation with the passage of time since reinforcement [27]). The levers were inserted into the chamber at the start of each trial and were withdrawn during the intertrial interval. Reinforcers were delivered only for responses on lever A during the first 25 s, and on lever B in the last 25 s of the trials. The positions of levers A and B (left versus right) were counterbalanced across subjects. The remaining 4 trials in each session were probe trials in which no reinforcers were delivered, which were interspersed randomly among the standard trials, with the constraint that at least one standard trial occurred between successive probe trials. Switching between the levers was restricted to one switch per trial: in each trial, the first response on lever B resulted in withdrawal of lever A until the start of the next trial [28–30].

*Yoked VI 30-s schedule (VI 30-s)* (*n* = 12). Each session consisted of 50 50-s trials – 46 standard VI trials and 4 probe trials in which no reinforcers were delivered. At the start of each trial, lever A was introduced into the chamber; when the ‘master’ rat in the FOPP group switched from lever A to lever B, lever A was withdrawn from the yoked rat's chamber and was replaced by lever B. A constant-probability VI 30-s [27] was operative continuously throughout the standard trials.

### 2.4 Immunohistochemistry

Ninety minutes after the final session the rats were deeply anaesthetized with pentobarbitone and perfused transcardially with phosphate-buffered physiological saline (PBS) (0.1 M) followed by 4% paraformaldehyde in PBS (formol PBS). Brains were removed and fixed in formol PBS. After 4 h, they were transferred to a 30% sucrose solution for 48 h. Forty-micrometer-thick coronal sections were cut on a freezing microtome. Free-floating sections were washed in PBS and then treated with 0.3% H_2_O_2_ in PBS for 30 min. Subsequently, the sections were treated with a blocking solution containing 3% normal goat serum (NGS) and 0.3% Triton-X in PBS, and incubated for 2 days at 4 °C with the primary antibody [polyclonal anti-Fos protein raised in rabbit (Santa Cruz Biotechnology, Santa Cruz, CA, USA), diluted 1:5000 in PBS containing 3% NGS and 0.3% Triton-X]. This was followed by incubation in the secondary antibody, biotinylated goat anti-rabbit immunoglobulin G (Vector Laboratories, Burlingame, CA, USA; 1:600), for 2 h, and by incubation with peroxidase-conjugated avidin–biotin complex (Vector Laboratories) for 1 h. The reaction was developed with 3,3′-diaminobenzidine. The sections were mounted on chrome-gelatin-coated microscope slides and dehydrated in graded alcohols (70%, 80%, 90%, and 100% ethanol), cleared in xylene and coverslipped with DPX.

Fos-positive nuclei were identified by the dark reaction product confined to the nucleus and quantified from digital images of sections at a magnification of 50× using ImageJ software (Wayne Rasband, National Institutes of Health, USA). The brain structures were outlined according to Paxinos and Watson's stereotaxic atlas [31]. The areas analysed were the following: infralimbic (ILPFC), prelimbic (PLPFC) and orbital (OPFC) prefrontal cortex, the dorsomedial (DMCP) and dorsolateral (DLCP) caudate-putamen, and three sub-regions of the nucleus accumbens (Acb) – the core (AcbC) and the medial and lateral portions of the shell (AcbS).

### 2.5 Data analysis

#### 2.5.1 Behavioural data

The mean response rate on each lever, *R*_A_ and *R*_B_, in successive 5-s time-bins of the probe trials was used to calculate the relative response rate on lever B, %*B* (100 × *R*_B_/[*R*_A_ + *R*_B_]). A two-parameter logistic function was fitted to each rat's relative response rate data: %*B* = 100/(1 + [*t*/*T*_50_]^ɛ^), where *t* is time from trial onset, *T*_50_ is the time at which %*B* = 50%, and *ɛ* is the slope of the function. The limen was defined as half the difference between *T*_75_ and *T*_25_ (*T*_75_ and *T*_25_ being the values of *t* corresponding to %*B* = 75% and %*B* = 25%), and the Weber fraction was calculated as the ratio of the limen to *T*_50_. Goodness of fit of the logistic functions was expressed as *r*^2^*.* The values of *T*_50_ and the Weber fraction, the overall response rate, and the overall reinforcement rate obtained in the entire session, were compared between the two groups using Student's *t*-test.

#### 2.5.2 Immunohistochemical data

Fos-positive nuclei were identified as described above. The number of Fos-positive nuclei in a standard area within each brain region was compared between groups by multivariate analysis of variance (MANOVA). Effect size was assessed using partial eta-squared, *η*_*p*_^2^. The areas used for quantifying Fos expression are shown in Fig. 3.

A significance criterion of *p* < 0.05 was used in all statistical analyses.

## 3 Results

### 3.1 Behavioural data

**Fig. 1:**
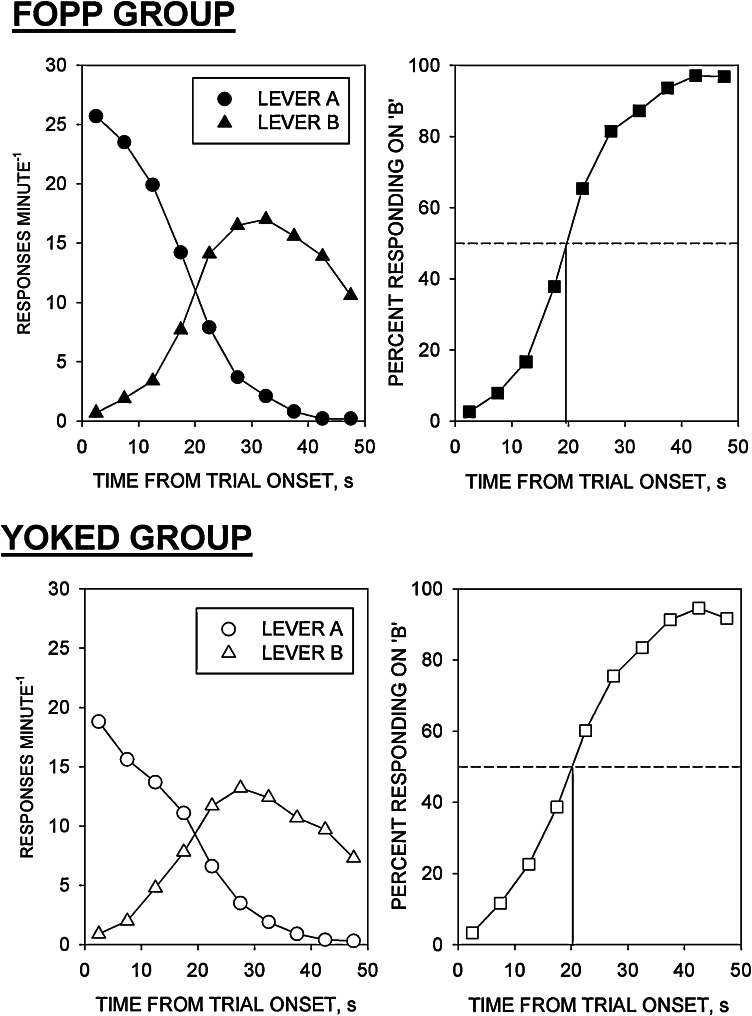
Comparison of performance on the free-operant psychophysical procedure (FOPP: upper graphs) and the yoked variable-interval schedule (YOKED: lower graphs). *Left-hand graphs*: absolute response rates on levers A (*descending curves*) and B (*ascending curves*); *ordinate*, response rate (responses min^−1^); *abscissa*, time from trial onset (s). *Right-hand graphs*: relative response rate on lever B (“psychometric function”); *ordinate*, percent responding on lever B; *abscissa*, time from trial onset (s). Points indicate group mean data.

The data from both groups are shown in Fig. 1. In both groups, response rate on lever A declined and response rate on lever B increased during the course of the trial. Relative responding on lever B increased monotonically as a function of time from trial onset. The behavioural indices derived from the two groups are shown in Table 1. Overall response rate was somewhat higher in the FOPP group than in the yoked VI group; however, this difference was not statistically significant [*t*(22) = 0.8, *N.S.*]. Neither the overall reinforcement rate [*t*(22) = 1.5, *N.S.*] nor the parameters of the logistic functions differed significantly between the groups [*T*_50_: *t*(22) = 0.2, *N.S.*; Weber fraction: *t*(22) = 1.2, *N.S.*].

### 3.2 Immunohistochemical data

**Fig. 2:**
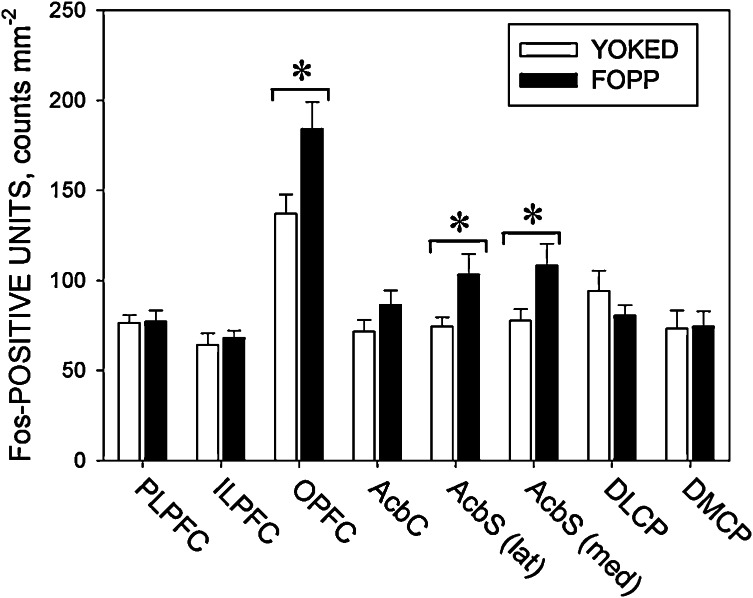
Density of Fos-positive units (counts.mm^−2^) counted in the cortical and striatal regions: infralimbic (ILPFC), prelimbic (PLPFC) and orbital (OPFC) prefrontal cortices, the nucleus accumbens core (AcbC) and medial and lateral portions of the nucleus accumbens shell (AcbS), and the dorsomedial (DMCP) and dorsolateral (DLCP) caudate-putamen. Columns show the group mean data (+SEM) in each area for the rats trained under the free-operant psychophysical procedure (FOPP: black columns) and the yoked variable-interval schedule (YOKED: white columns). Significant difference between the groups, *p < 0.05.

**Fig. 3:**
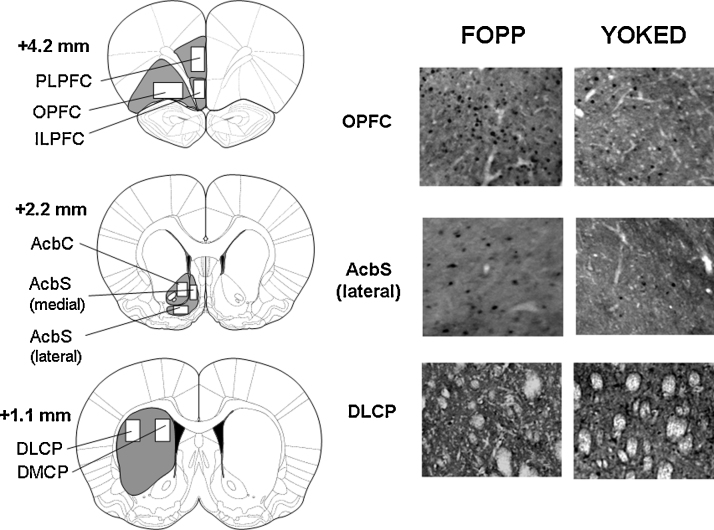
*Left-hand column.* Diagrammatic representations of the areas selected for counting Fos-positive units (AP locations, measured from bregma, as indicated; modified from Paxinos and Watson [31]). ILPFC: infralimbic prefrontal cortex; PLPFC: prelimbic prefrontal cortex; OPFC: orbital prefrontal cortex; AcbC: core of nucleus accumbens; AcbS: shell of nucleus accumbens; DLCP: dorsolateral caudate-putamen; DMCP: dorsomedial caudate-putamen. *Middle and right-hand columns*. Examples of Fos expression in the OPFC, the lateral AcbS and the DLCP in a representative rat trained under the free-operant psychophysical procedure (FOPP: middle column) and a rat trained under the yoked variable-interval schedule (YOKED: right-hand column).

Fig. 2 shows the group mean (+S.E.M.) numbers of Fos-positive units detected in the eight brain regions examined. Fos expression was significantly greater in the FOPP group than in the yoked VI group in the OPFC [*F*(1, 22) = 6.5, *p* < 0.05; *η*_*p*_^2^ = 0.23] and the lateral [*F*(1, 22) = 5.6, *p* < 0.05; *η*_*p*_^2^ = 0.20] and median AcbS [*F*(1, 22) = 5.2, *p* < 0.05; *η*_*p*_^2^ = 0.19]. Fos expression was somewhat greater in the AcbC of the FOPP group than the yoked VI group, although this difference fell short of statistical significance [*F*(1, 22) = 2.1, *NS*; *η*_*p*_^2^ = 0.09]. No significant differences in Fos expression were seen in the ILPFC, PLPFC, DMCP or DLCP [*F*(1, 22) ≤ 1.1, *NS*; *η*_*p*_^2^ < 0.05, in each case]. Representative examples of coronal sections showing Fos expression in the OPFC, lateral AcbS and DLCP of a rat from each experimental group are given in Fig. 3.

## 4 Discussion

Performance on the FOPP was similar to that reported in many previous studies [17,18,28–30,32–45]. Relative response rate (%*B*) increased monotonically towards 100% during the course of the trial, the two-parameter logistic function providing a good description of the psychometric data derived from individual subjects. In agreement with several previous reports, *T*_50_ (mean = 19.4 s) occurred somewhat earlier in the trial than the point at which reinforcer allocation was transferred from lever A to lever B (25 s) [34–42].

The purpose of the yoked VI procedure was to provide a control group that was exposed to a schedule that did not entail temporal differentiation of responding, yet was well matched to the FOPP in terms of non-temporal variables such as food deprivation level, switching between operanda, reinforcement rate and overall response rate. The two groups underwent identical food deprivation regimens and were trained in identical operant conditioning chambers. Switching from lever A to lever B was restricted to one switch per trial in both groups. Overall response rates were somewhat lower in the yoked VI group than in the FOPP group; however the difference between the two groups was not statistically significant. There was no significant difference between the rates of reinforcement in the two groups. It is also noteworthy that the psychometric functions derived for the rats trained under the two procedures yielded similar values of *T*_50_ and the Weber fraction. These data indicate a satisfactory degree of matching of the two groups with respect to these behavioural measures.

The density of Fos-positive neurones (counts mm^−2^) in the OPFC and the medial and lateral sub-regions of the AcbS was greater in the rats exposed to the FOPP than in those exposed to the yoked VI schedule, suggesting a greater activation of these areas during the performance of the former task. This difference in Fos expression between the two groups is unlikely to have been caused by differences in reinforcer consumption, repetitive execution of the operant response or the occurrence of switching from lever A to lever B, because the two groups were reasonably well matched with respect to these variables (see above). The present results are thus consistent with the notion that neuronal activation in the OPFC and AcbS was related to the temporal control of behaviour exerted by the FOPP. It should be noted, however, that although the main difference between the two tasks is the presence of temporal control over the distribution of responding in the case of the FOPP, a yoked procedure such as that used in the present experiment cannot control for all potentially relevant factors [46]. For example, the retraction of lever A is a response-contingent event in the FOPP, whereas in the yoked procedure the replacement of lever A by lever B was independent of the rat's behaviour. These and other factors need to be explored in future experiments (see below).

The present results obtained with the FOPP are concordant with the findings of a previous experiment in which enhanced Fos expression in the OPFC and Acb was associated with temporal discrimination performance in retrospective timing schedules [11]. However, in contrast to the findings of Valencia-Torres et al. [11] with retrospective timing tasks, a recent experiment by the same authors [15] found enhancement of Fos expression in the OPFC but not in the Acb following exposure to the fixed-interval peak procedure. These authors suggested that the difference between the results of their two studies could have reflected the use of an immediate timing task (the fixed-interval peak procedure) in the latter experiment, as opposed to the retrospective timing tasks used in the earlier one [15]. The present results cast doubt on this explanation because the FOPP, like the fixed-interval peak procedure, is regarded as an immediate timing schedule [1,2]. One feature that the FOPP has in common with the retrospective timing schedules used by Valencia-Torres et al. [11] which is not shared by the fixed-interval peak procedure is the provision of two operanda. The FOPP, like the retrospective timing tasks, entails explicit choice between two operanda, in contrast to the fixed-interval peak procedure in which timing is assessed from variation of response rate on a single operandum. It is possible that the activation of the ventral striatum during performance of some, but not all, interval timing tasks reflects the engagement of this structure in time-based choice behaviour. Further experiments employing a wider range of timing schedules are needed to investigate this possibility. For example, it would be of interest to examine the pattern of Fos expression in rats trained on discrete-trials temporal generalization tasks [2]. The present suggestion that the ventral striatum may be engaged specifically by timing schedules that entail choice between operanda implies that no enhancement of Fos expression should occur following exposure to this type of single-operandum retrospective timing task.

Another question that may be addressed in future experiments is the possible role of cortical and striatal activation in response suppression or ‘inhibition’. It is known that the acquisition of accurate timing performance on the FOPP entails learned suppression of switching from lever A to lever B [28]. This is a potentially relevant difference between the FOPP and the yoked VI schedule used in the present experiment, since switching was not under temporal control in the yoked procedure. However, it seems unlikely that this difference could account for the different patterns of enhanced Fos expression seen with different types of timing task, because there is no obvious role for response suppression in performance on the retrospective timing schedules that were associated with enhanced Fos expression in the Acb [11], whereas there is evidence that learned suppression of responding occurs in the acquisition of accurate timing on the fixed-interval peak procedure [47], which was found not to be associated with enhanced Fos expression in this structure [15].

Another type of choice task in which timing processes may be involved is inter-temporal choice, in which organisms choose between reinforcers that differ with respect to size and delay [48]. The hypothetical process of delay discounting, whereby reinforcing outcomes are deemed to be devalued by delays interposed between the choice response and the delivery of the primary reinforcer, is widely assumed to imply the operation of a timing process [49–52]. Indeed, inter-temporal choice tasks have been classified as ‘prospective timing schedules’ [1,2]. It is of interest, therefore, that there is considerable evidence for a role of the prefrontal cortex and ventral striatum in inter-temporal choice [53–55]. For example, lesions of the OPFC [56,57] and Acb [58–62] have been found to increase the rate of delay discounting, and performance of inter-temporal choice tasks has been found to produce enhanced Fos expression in both the OPFC and the Acb [63].

Although, as suggested above, the involvement of the ventral striatum in interval timing may be restricted to timing tasks that entail explicit choice, this does not imply that the ventral striatum plays a pivotal role in all choice situations. Indeed, it has been found that while choice between reinforcers differing only in delay resulted in enhanced Fos expression in the ventral striatum, choice between reinforcers differing only in magnitude did not [63]. Moreover, the enhancement of Fos expression in the ventral striatum seen following exposure to retrospective timing schedules was not seen following exposure to light intensity discrimination schedules based on the same discrete-trials choice contingencies as the timing tasks [11].

It is unclear whether the same areas of the ventral striatum are involved in performance on prospective timing schedules as in performance on retrospective and immediate timing schedules. Valencia-Torres et al. [11] found that the enhancement of Fos expression that accompanied performance on retrospective timing schedules was most pronounced in the AcbS, although the neighbouring AcbC was also affected. Most of the evidence for the involvement of the ventral striatum in inter-temporal choice has focused on the AcbC [58–62], although there do not appear to have been any studies that have directly compared the roles of the AcbC and AcbS in inter-temporal choice [for review, see 53–55].

In contrast to the ventral striatum, the OPFC's role in interval timing appears not to be restricted to choice-based tasks, as enhanced Fos expression has been found in this area following exposure to the single-operandum fixed-interval peak procedure [15]. In the case of inter-temporal choice, the role of the OPFC is not restricted to delay discounting; lesions of this area may alter inter-temporal choice by reducing the incentive value of food reinforcers as well as by increasing the rate of delay discounting [58]. Moreover, levels of Fos expression in the OPFC are enhanced following exposure to choice between reinforcers of different magnitudes, as well as choice between reinforcers of different delays [63].

In summary, the results of this experiment implicate the OPFC and the ventral striatum in the control of timing performance on the FOPP. It is suggested that the OPFC may be involved in the performance of a wide range of timing tasks, whereas the ventral striatum may be engaged more specifically in those timing schedules that entail operant choice.

## Figures and Tables

**Table 1:** Experiment 1: Behavioural indices.

Behavioural index^a^	Group mean data (±S.E.M.)	
	FOPP group	Yoked group
*T*_50_ (s)	19.4 ± 1.0	19.8 ± 1.5
Weber fraction	0.278 ± 0.037	0.362 ± 0.056
*r*^2^	0.958 ± 0.008	0.961 ± 0.008
Overall response rate (responses min^−1^)	53.2 ± 4.3	46.3 ± 6.7
Reinforcement rate (reinforcers session^−1^)	49.8 ± 0.6	51.1 ± 0.6

a Indices were derived from performance in probe trials, except for reinforcement rate, which was derived from performance in standard trials.
